# Assessment of pre-extubating recurrent laryngeal nerve palsy using ultrasound in postoperative patients with esophageal cancer: a prospective observational study

**DOI:** 10.1007/s00540-024-03315-7

**Published:** 2024-03-02

**Authors:** Tomomi Kaneko, Takao Kato, Yuki Shiko, Yohei Kawasaki, Kaoru Koyama

**Affiliations:** 1 Department of Anesthesiology, Saitama Medical Center, Saitama Medical University, 1981 Kamoda, Kawagoe-city, Saitama 350-8550 Japan; 2 Biostatistics Section, Clinical Research Center, Chiba University Hospital, Chiba, Japan; 3 Faculty of Nursing, Japanese Red Cross College of Nursing, Tokyo, Japan

**Keywords:** Recurrent nerve palsy, Vocal cords, Ultrasound, Esophageal cancer, Surgery

## Abstract

**Purpose:**

Ultrasound performed after extubation has been suggested to be useful for the diagnosis of recurrent laryngeal nerve (RLN) paralysis. However, the use of ultrasound for this purpose before extubation has not been examined. The aim of this study was to examine the versatility (interrater reliability) and usefulness of ultrasound for evaluating the movement of vocal cords before extubation.

**Methods:**

The subjects were 30 patients who underwent radical surgery for esophageal cancer from August 2020 to December 2021. An experienced examiner performed an ultrasound examination before and after elective extubation on the day after surgery to evaluate RLN paralysis and record videos. Bronchoscopy was then performed to make a definite diagnosis. Three anesthetists blinded to the diagnosis also evaluated the cases using the videos, and the versatility of the examination was determined using a kappa test.

**Results:**

The diagnostic accuracies of the examiner and three anesthetists were 76.7%, 50.0%, 53.3%, and 46.7%, respectively, and the kappa coefficients for the examiner with the anesthetists were 0.310, 0.502, and 0.169, respectively. The sensitivity, specificity, positive predictive value and negative predictive value for diagnosis of RLN paralysis by the examiner using ultrasound before extubation were 0.57, 0.95, 0.80, and 0.87, respectively.

**Conclusion:**

These results indicate a lack of versatility of the ultrasound examination based on the low kappa coefficients. However, with an experienced examiner, ultrasound can serve as a non-invasive examination that can be performed before extubation with high accuracy and specificity for diagnosis of postoperative RLN paralysis.

**Supplementary Information:**

The online version contains supplementary material available at 10.1007/s00540-024-03315-7.

## Introduction

Recurrent laryngeal nerve (RLN) paralysis develops as a complication of lymph node dissection around the RLN in esophageal cancer surgery, with reported incidences of 10–32% [1, 2] and as high as 59.5% [3]. Development of RLN paralysis is accompanied by high risks of hoarseness and aspiration [3–6]. Definitive diagnosis is usually made by observing the movement of the vocal cords with a laryngoscope or bronchoscope [1–8], but the use of these instruments may cause discomfort to the patient [4–8].

Recent reports have suggested that transcutaneous laryngeal ultrasonography (TLUSG) is useful for evaluation of the vocal codes [4–8]. Ultrasonography can be performed at the bedside as a non-invasive examination with no complications [4]. However, ultrasonography for evaluation of paralysis of the vocal codes has mainly been performed after extubation, with only one case report describing the diagnosis of bilateral vocal cord paralysis before extubation [9]. Diagnosis of vocal cord paralysis before extubation would allow anticipation of the need for reintubation or tracheostomy and adequate preparation in the event of paralysis. Therefore, the current study was performed to evaluate the versatility and diagnostic value of ultrasound for the evaluation of movement of the vocal cords before extubation.

## Methods

This study was performed after obtaining approval from the ethical committee of Saitama Medical Center, Saitama Medical University (approval No.: 2381). The checklist for Strengthening the Reporting of Observational Studies in Epidemiology (STROBE) was used. The subjects were patients who underwent radical surgery for esophageal cancer (subtotal esophagectomy/gastric tube reconstruction/two- or three-field dissection) at Saitama Medical Center from July 2020 to December 2021. Patients who underwent surgery for esophageal cancer other than radical surgery, those with extubation in the operation room, and those who did not agree to participate in the study were excluded. Before surgery, the objectives of the study were explained to all subjects and written informed consent was obtained.

The primary outcome was an examination of the versatility (interrater reliability) of the ultrasound examination using a kappa coefficient. The secondary outcomes were a comparison of the evaluation of vocal cord movements by ultrasound before extubation with that by bronchoscopy after extubation, the accuracy of evaluations of RLN paralysis made by the examiner and three anesthetists; comparison of evaluation by ultrasound after extubation with that by bronchoscopy; evaluation with intraoperative neurophysiological monitoring (IONM) after extubation; confirmation of cannulation of the cricothyroid membrane after extubation; and postoperative complications. The required number of subjects was estimated to be 30 to achieve an accuracy with a 95% confidence interval (CI) of 0.1 based on kappa = 0.6 (standard deviation [SD]: 0.138), which was assumed based on a preliminary study.

General anesthesia was achieved with epidural anesthesia, and intubation using a special tube (NIM TriVantage EMG Tube®, Medtronic plc, Dublin, Ireland) was used to conduct IONM. Intraoperative one-lung ventilation was performed using a bronchial blocker (TBC Bronchial Blocker®, Fuji Systems, Tokyo, Japan). The same surgeon performed the surgery for all 30 subjects. After surgery, the patient was transferred to the intensive care unit (ICU) after the replacement of the tube to a suction above cuff endotracheal tube (SACETT®, Suction Above Cuff Endotracheal Tube, Smiths Medical, Plymouth, MN, USA). An artificial ventilator was used on the day of surgery and extubation was performed the next morning. Dexmedetomidine was used for sedation in the ICU and the Richmond Agitation-Sedation Scale (RASS) was −1 to 0 upon extubation. The sedation level did not change from before to after extubation, and the patient was able to perform indicated actions such as deep breathing. Before extubation, the artificial ventilator was set at continuous positive airway pressure (CPAP) with positive end-expiratory pressure (PEEP) at 8 cmH_2_O and pressure support (PS) at 5 cmH_2_O.

**Fig. 1:**
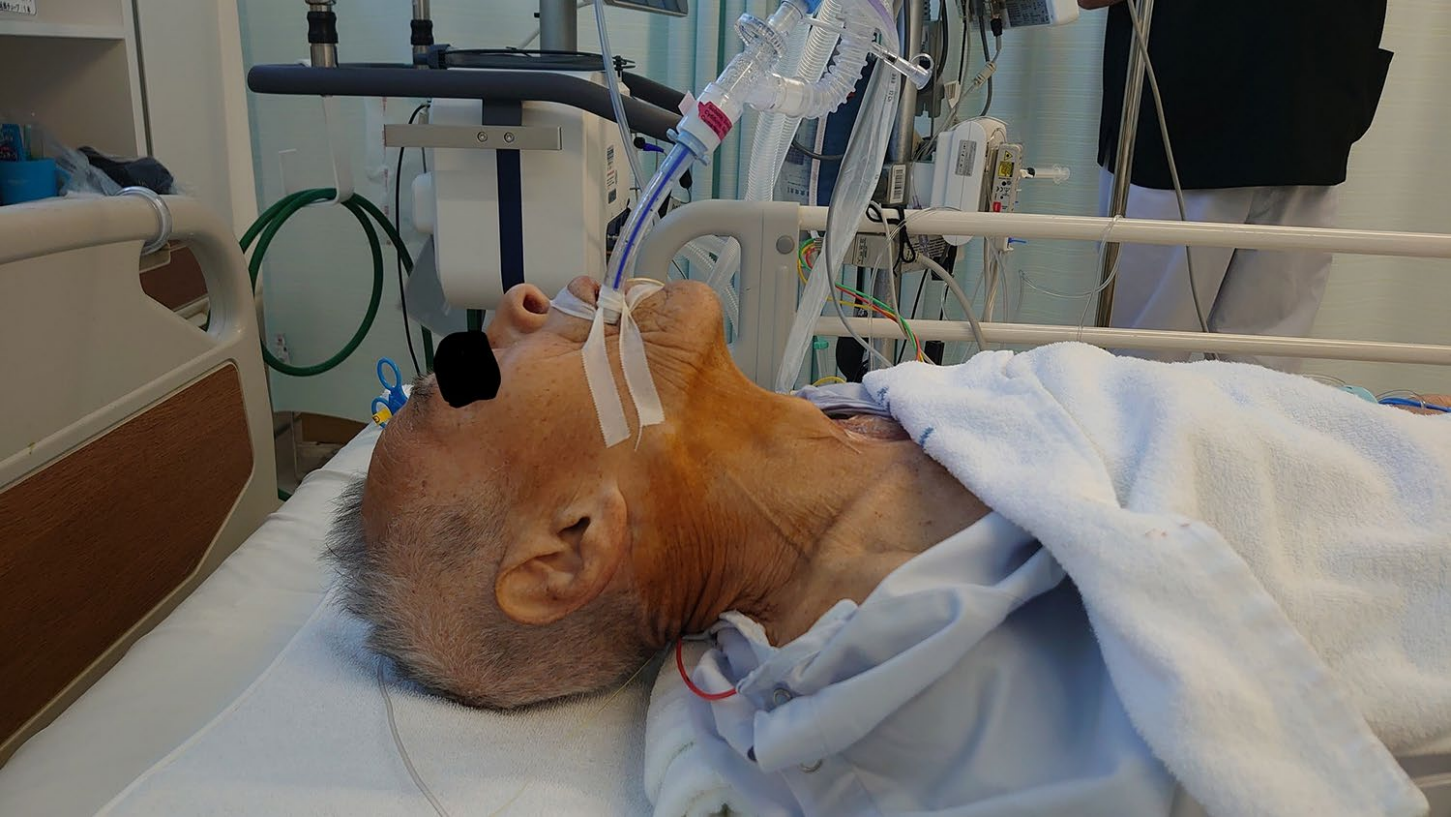
Patient position. This photograph was taken with the patient’s permission. The examination was performed with the patient in a supine position with the neck extended using a shoulder pillow

**Fig. 2:**
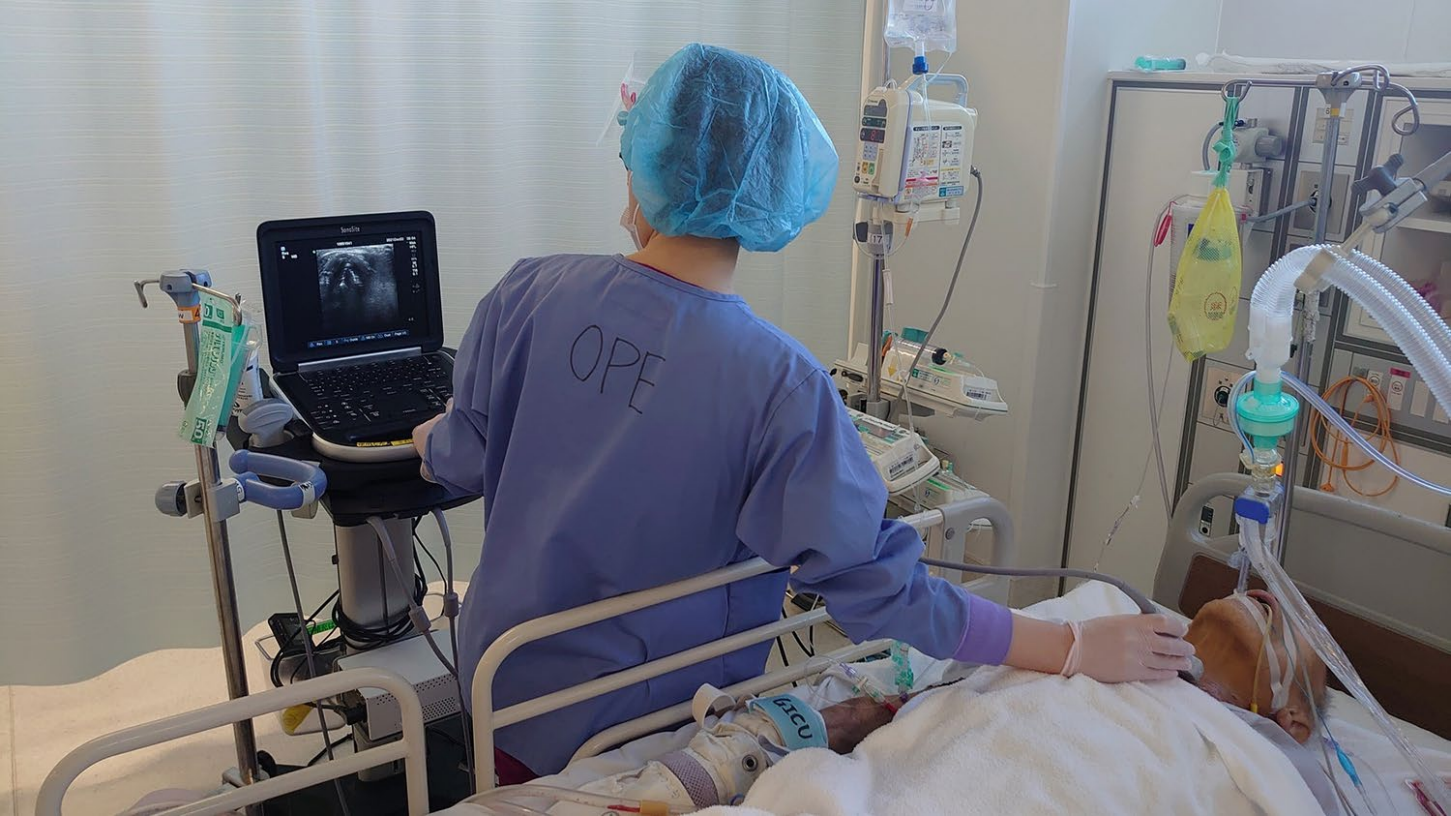
Filming scene before extubation. Dexmedetomidine was used for sedation and RASS was −1 to 0 upon extubation. The artificial ventilator was set at CPAP with PEEP at 8 cmH_2_O and PS at 5 cmH_2_O. The examiner performed the test while instructing the patient on the timing of breathing. *RASS* Richmond agitation-sedation scale, *CPAP* continuous positive airway pressure, *PEEP* positive end-expiratory pressure, *PS* pressure support.

Immediately before and after extubation, movements of the vocal cords were evaluated using ultrasound equipment (SonoSite Edge II®, 6–13 Hz, Linear Probe, FUJIFILM, Tokyo, Japan). The results were recorded as “with paralysis”, “without paralysis” or “unevaluable.” As shown in Figs. 1 and 2, the examination was performed with the patient in a supine position with the neck extended. Paralysis of the vocal cords was subsequently evaluated using a bronchoscope (Ambu® aScope™ 4 Broncho Slim 3.8/1.2, Ambu, Ballerup, Denmark) by the surgeon who performed the esophageal cancer surgery and the results were recorded using the same categories. The surgeon was blinded to the ultrasound findings before extubation. Only a case with complete paralysis was diagnosed as paralyzed. The examiner in all of these evaluations had experience of ultrasound examination for 33 patients in a previous study.

A fourfold table was prepared to examine the sensitivity, specificity, positive predictive value (PPV), and negative predictive value (NPV) of each examination. To examine the versatility of the ultrasound examination, three anesthetists evaluated the ultrasound videos on another day, again using the same categories. Versatility was evaluated using a kappa coefficient. The examiner and three anesthetists were not involved in the perioperative management of the subjects and were blinded to the IONM results. The frequency and ratio were obtained for nominal and ordinal variables, and the mean and standard deviation were calculated for continuous variables. The main outcome was evaluated using a kappa test. All calculations were performed in SAS v.9.4 (SAS Institute, Cary, NC, USA). There was no adjustment for bias.

## Results

**Fig. 3:**
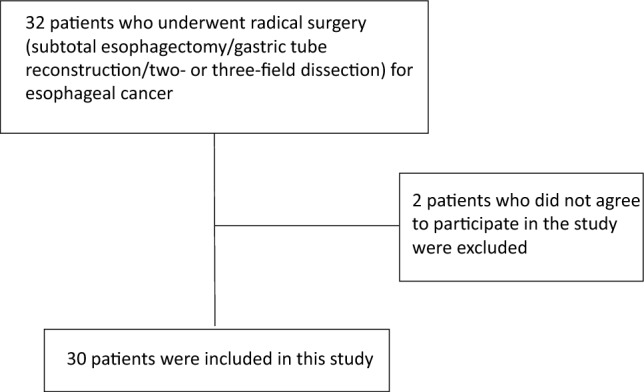
Flowchart of inclusion and exclusion of cases. Of 32 patients who underwent radical surgery for esophageal cancer from July 2020 to December 2021, 2 who did not agree to participate in the study were excluded

Of 32 patients who underwent radical surgery for esophageal cancer from July 2020 to December 2021, 2 who did not agree to participate in the study were excluded (Fig. 3). The background of the 30 subjects included in the analysis is shown in Table 1.

**Table 1:** Backgrounds of patients with and without paralysis

Item	With paralysis (*n* = 7)	Without paralysis (*n* = 23)	Total (*n* = 30)
Sex (male) (%)	7 (100)	18 (78.3)	25 (83.3)
Age (years)	72.7 (6.65)	69.8 (8.18)	70.5 (8.08)
Height (m)	1.66 (0.09)	1.63 (0.10)	1.64 (0.09)
Weight (kg)	60.9 (8.81)	59.9 (12.6)	60.1 (11.7)
Body mass index (kg m^−2^)	22.1 (1.99)	22.5 (4.62)	22.4 (4.27)
Surgery			
Thoracoscopic subtotal esophagectomy	5 (71.4)	11 (47.8)	16 (69.6)
Subtotal esophagectomy with thoracolaparotomy	2 (28.6)	11 (47.8)	13 (56.5)
Resection of the cervical esophagus	0 (0)	1 (4.3)	1 (4.3)
Preoperative complications			
Hypertension	4 (57.1)	10 (43.5)	14 (60.9)
Smoker	6 (85.7)	18 (78.3)	24 (80.0)
Diabetes	1 (14.3)	2 (8.7)	3 (10.0)
ASA			
1	0 (0)	0 (0)	0 (0)
2	6 (85.7)	18 (78.3)	24 (80.0)
3	1 (14.3)	5 (21.7)	6 (20.0)
Methods of anesthesia			
Air-O_2_-Sevoflurane (AOS)	3 (42.9)	10 (43.5)	13 (43.3)
Total intravenous anesthesia (TIVA)	4 (57.1)	13 (56.5)	17 (56.7)
Concomitant use of epidural anesthesia	5 (71.4)	22 (95.7)	27 (90.0)

Comparison between the groups was not performed due to the small number of subjects. Age, height, weight, and BMI are shown as mean (SD). All other data are shown as n (%)

*ASA-PS* American Society of Anesthesiologists Physical Status

Subjects identified with RLN paralysis in each examination (Table 2–4) were categorized as positive. Postoperative RLN paralysis was confirmed in a total of 7 subjects (23%), of whom 2 underwent ablation of the left RLN upon lymph node dissection. Compared with diagnoses by bronchoscopy, of the 28 evaluable patients using ultrasound before extubation, 4 of 7 and 20 of 21 were diagnosed with and without paralysis, respectively (Table 2); of the 26 evaluable patients in ultrasound after extubation, 1 of 4 and 22 of 22 were diagnosed with and without paralysis, respectively (Table 3); and of the 29 evaluable patients using IONM, 5 of 6 and 21 of 23 were diagnosed with and without paralysis, respectively (Table 4).

**Table 2:** Ultrasound evaluation before extubation

		Bronchoscopy		
		With paralysis	Without paralysis	Total
Ultrasound before extubation	With paralysis	4	1	5
	Without paralysis	3	20	23
	Total	7	21	28^a^

^a^ Two of the 30 subjects could not be evaluated

**Table 3:** Ultrasound evaluation after extubation

		Bronchoscopy		
		With paralysis	Without paralysis	Total
Ultrasound after extubation	With paralysis	1	0	1
	Without paralysis	3	22	25
	Total	4	22	26^a^

^a^ Four of the 30 subjects could not be evaluated, including one subject who could also not be evaluated using ultrasound before extubation

**Table 4:** Evaluation using intraoperative neurophysiological monitoring (IONM)

		Bronchoscopy		
		With paralysis	Without paralysis	Total
IONM	With paralysis	5	2	7
	Without paralysis	1	21	22
	Total	6	23	29^a^

^a^ IONM was not performed in one of the 30 subjects

The sensitivity, specificity, PPV, and NPV for each diagnostic method are shown in Table 5. These respective values were 0.57 (95%CI: 0.20–0.94), 0.95 (95%CI: 0.86–1.00), 0.80 (95%CI: 0.45–1.00), and 0.87 (95%CI: 0.73–1.00) using ultrasound before extubation; 0.25 (95%CI: 0.00–0.67), 1.0 (95%CI: 1.00–1.00), 1.0 (95%CI: 1.00–1.00), and 0.88 (95%CI: 0.75–1.00) with ultrasound after extubation; and 0.83 (95%CI: 0.54–1.00), 0.91 (0.80–1.00), 0.71 (95%CI: 0.38–1.00), and 0.95 (95%CI: 0.87–1.00) using IONM (Table 5).

**Table 5:** Sensitivity, specificity, positive predictive value (PPV), and negative predictive value (NPV) in ultrasound examinations and IONM

Item	Ultrasound before extubation	Ultrasound after extubation	IONM
Sensitivity (95% CI)	0.57 (0.20–0.94)	0.25 (0.00–0.67)	0.83 (0.54–1.00)
Specificity (95% CI)	0.95 (0.86–1.00)	1.00 (1.00–1.00)	0.91 (0.80–1.00)
PPV (95% CI)	0.80 (0.45–1.00)	1.00 (1.00–1.00)	0.71 (0.38–1.00)
NPV (95% CI)	0.87 (0.73–1.00)	0.88 (0.75–1.00)	0.95 (0.87–1.00)

*IONM* intraoperative neurophysiological monitoring

The kappa values were 0.310 (95%CI: −0.007 to 0.628), 0.502 (95%CI: 0.204–0.801), and 0.169 (95%CI: −0.032 to 0.426), respectively, showing low rates of concordance. The accuracy rate before extubation was 76.7% for the examiner, while those of the three anesthetists were 50.0%, 53.3%, and 46.7%, respectively.

Postoperative complications are shown in Table 6. Many cases of hoarseness and pneumonia occurred in patients with paralysis. Minitracheostomy for expectoration was performed for two patients who showed no paralysis.

**Table 6:** Postoperative complications

Complication	With paralysis	Without paralysis	Total
Hoarseness	3 (42.9)	1 (4.3)	4 (13.3)
Minitracheostomy	0 (0)	2 (8.7)	2 (6.7)
Pneumonia	2 (28.6)	1 (4.3)	3 (10.0)

Data are shown as the number of subjects (%)

## Discussion

The primary outcome of concordance among the examiner and the three anesthetists was low based on the generally accepted value for a kappa coefficient of ≥ 0.6 to show high concordance [10]. This suggests a lack of versatility in the examination. Among the secondary outcomes, diagnosis using ultrasound before extubation showed relatively low sensitivity, but specificity, PPV, and NPV were high, and the accuracy of the examiner was also high (76.7%). For diagnosis after extubation, the sensitivity with ultrasound was lower than that before extubation, but specificity, PPV, and NPV were higher than those with IONM.

In this study, the versatility of the examination was not shown based on the low kappa coefficient. Many examinations using TLUSG are performed by otolaryngologists, whereas anesthetists have few opportunities to use TLUSG. Oliveira et al. concluded that experience of ≥ 20 cases is necessary to identify the cricothyroid membrane using ultrasound [11]. In our study, the examiner had experience with TLUSG for 33 patients in a previous study, whereas the three anesthetists who were asked to examine the videos did not perform TLUSG in daily practice and had no experience in viewing ultrasound images of the respiratory tract. In addition, the examiner could feel timing of intake and exhaled air when operating the ultrasound equipment, but the three anesthetists had no contact with the subjects. The accuracy rate of the examiner was 76.7%, while those of the three anesthetists were only 50.0%, 53.3%, and 46.7%. Thus, the low kappa coefficient may have been due to the lack of experience and reduced information available to the three anesthetists.

The diagnostic sensitivity, specificity, PPV and NPV before extubation were 0.57, 0.95, 0.80 and 0.87, respectively. A review by da Costa et al. [4]. found sensitivity, specificity, PPV and NPV in TLUSG after extubation ranging from 0.33, 0.95, 0.42 and 0.89, respectively, to 0.93, 0.98, 0.78 and 0.99, respectively [4]. In this review, the patients underwent surgery on the thyroid and included many young female patients. In contrast, our data obtained after esophageal cancer surgery included those for many elderly male patients. Some reports have suggested that evaluation of the vocal cords by TLUSG is more difficult in male, elderly or tall patients, and this might be a cause of the low sensitivity observed in our study [4, 12].

The ultrasound examination performed before extubation in this study had high PPV and NPV even when the range of the vocal cords was limited by an intubation tube. Invasive laryngoscopy and bronchoscopy are generally used to diagnose RLN paralysis, whereas evaluation with ultrasound is a non-invasive method that has high accuracy and specificity with an experienced operator. Thus, this is a useful screening method before extubation. The specificity, PPV and NPV in TLUSG after extubation were also high, but the sensitivity was low. After extubation, movements of the vocal cords were examined when a subject let out their voice, in addition to upon intake and exhaled air. A paralyzed vocal cord may appear normal when an unaffected vocal cord moves well in an ultrasound examination, and this might have caused lower sensitivity than that observed before extubation. In fact, the contralateral vocal cord in patients with paralysis moved well in our study. The sensitivity, specificity, PPV and NPV of 0.83, 0.91, 0.71 and 0.95, respectively, for diagnosis with IONM in this study did not differ significantly from those of 0.67, 0.96, 1.00 and 0.97, respectively, found by Kobayashi et al. using IONM [2]. PPV was slightly lower in the current study, which could be due to deviation of the intubation tube due to operation or a blocker inserted for one-lung ventilation, sputum or oral discharge, which might have affected the monitoring. In addition, IONM can only determine the presence or absence of a response to recurrent nerve stimulation and the strength of the response, and a slight deviation of the dedicated tube can markedly change the evaluation, making diagnosis of paralysis difficult. In contrast, TLUSG can visualize vocal cord movement and reproducibly determine paralysis as long as recurrent nerve stimulation is possible intraoperatively. However, during surgery, the patient was in the lateral recumbent position, which made it difficult to use the probe due to the limited range of manipulation. However, it may be possible to determine recurrent nerve palsy more simply than IONM by modifying the probe.

As shown in Table 6, hoarseness was not found in all patients with RLN paralysis and may occur in some patients without paralysis. RLN paralysis is highly correlated with a risk of postoperative pneumonia after esophageal cancer surgery [13]. Definite diagnosis by objective examination, such as bronchoscopy, enables early intervention for preventive treatment of pneumonia and improvement of the medium- to long-term prognosis. Since the beginning of 2020, the COVID-19 pandemic has caused many difficulties in the medical field, and examiners using bronchoscopy have a high risk of exposure to salivary splash [14]. However, TLUSG is a transdermal examination with no risk of such exposure. Although our results suggested that bronchoscopy cannot be switched to TLUSG immediately, a non-invasive examination with a low risk of exposure to medical staff that can be performed before extubation enables prediction of risks for respiratory failure immediately after extubation and reintubation, and may improve the short-term prognosis in an environment in which an unknown infection may develop at some time in the future. Ultrasound examination of the air duct may also be useful for the evaluation of the vocal cords and may be better than palpation to identify the cricothyroid membrane in the acute phase [15–17]. A certain level of experience is required for evaluation with TLUSG [11]. Thus, it is important that anesthetists and intensivists who are usually engaged in airway management become familiar with TLUSG in daily practice so that they can evaluate a patient immediately when required.

There are some limitations in this study. First, the number of subjects required was estimated assuming a high kappa coefficient based on preliminary and previous studies, but the kappa coefficient in the study was lower than we assumed. The lack of experience of the three evaluators in making video judgments in TLUSG may have affected the results. However, the previous study also included three anesthesiologists with no prior experience or training, and their pre-extubation kappa coefficients were 0.568, 0.668, and 0.578, respectively, which showed the versatility of the method. Therefore, we anticipated that the three anesthesiologists who judged the video could show the method’s versatility without prior experience. Second, evaluations could not be made for 2 and 4 subjects before and after extubation, respectively. In one case, evaluation was not possible by ultrasound before and after extubation. The causes could not be examined statistically due to the small number of subjects, but the patients who could not be evaluated were all male. Evaluation of the vocal cords with TLUSG has been suggested to be more difficult in male patients [4, 12] and this might be a factor in the unevaluable cases. However, key factors after extubation may differ from those affecting evaluation before extubation, and this issue requires further study. Third, we did not evaluate the laterality or position of the vocal cords. We focused on the presence or absence of paralysis, as in previous studies, and did not evaluate details such as the laterality or whether the position of vocal cord paralysis was lateral or median [5–8, 12]. However, when the paralyzed vocal cords are positioned laterally, airway protection is compromised and the risk of aspiration is increased [3–6]. On the other hand, if the paralyzed vocal cords are located medially, the airway is narrowed and dyspnea may be present, even if only on one side [18]. Therefore, it is very important to evaluate the position of the vocal cords [19]. The laterality is also important. In general, vocal cord paralysis after esophageal cancer surgery and tracheal intubation is more common on the left side [1, 3, 20], and such left-sided vocal cord paralysis is reported to have a worse prognosis than right-sided paralysis [3]. Although it was not possible to evaluate the laterality and location of paralysis in this study, we will further investigate the possibility of using ultrasound to evaluate the laterality and location of paralysis in the future.

## Conclusions

The versatility of ultrasound for the detection of RLN paralysis could not be confirmed, but TLUSG before extubation may be useful if an experienced examiner makes the diagnosis, as seen after extubation. Use of ultrasound in daily practice by anesthetists and intensivists engaged in the management of the airway is likely to increase the versatility of TLUSG in the future.

### Supplementary Information

Below is the link to the electronic supplementary material.

Supplementary file1 (MP4 3917 KB)

Supplementary file2 (MP4 3974 KB)

## Data Availability

All data generated or analyzed during this study are included in this published article and its supplementary information files.
